# Building phonetic categories: an argument for the role of sleep

**DOI:** 10.3389/fpsyg.2014.01192

**Published:** 2014-10-28

**Authors:** F. Sayako Earle, Emily B. Myers

**Affiliations:** ^1^Department of Speech, Language, and Hearing Sciences, University of Connecticut, Storrs, CT, USA; ^2^Department of Psychology, University of Connecticut, Storrs, CT, USA; ^3^Haskins Laboratories, New Haven, CT, USA

**Keywords:** speech perception, learning, memory, consolidation, phonology

## Abstract

The current review provides specific predictions for the role of sleep-mediated memory consolidation in the formation of new speech sound representations. Specifically, this discussion will highlight selected literature on the different ideas concerning category representation in speech, followed by a broad overview of memory consolidation and how it relates to human behavior, as relevant to speech/perceptual learning. In combining behavioral and physiological accounts from animal models with insights from the human consolidation literature on auditory skill/word learning, we are in the early stages of understanding how the transfer of experiential information between brain structures during sleep manifests in changes to online perception. Arriving at the conclusion that this process is crucial in perceptual learning and the formation of novel categories, further speculation yields the adjacent claim that the habitual disruption in this process leads to impoverished quality in the representation of speech sounds.

## INTRODUCTION

Categorical perception refers to the phenomenon by which listeners demonstrate non-linear perception of items across an acoustic-phonetic continuum. In speech, we observe this behaviorally in the more accurate discrimination of two different phonetic tokens lying across a category boundary, compared to two phonetic tokens of a comparable acoustic distance occurring within the boundaries of the same category (Liberman et al., 1957; see Repp and Liberman, 1987 for review). Phonological contrasts correspond to the sounds of speech that are capable of signaling change in the lexical meaning in the signal (Chomsky and Halle, 1968), and each phoneme encompasses many acoustically distinct phonetic tokens. During spoken language processing, listeners map incoming phonetic tokens to existing phonological representations. The formation of phonetic categories^1^ is mediated by different stages of memory processing, which encode one’s experience with novel speech sounds and lead to generalization away from the context of that initial encounter. Thus, the focus of this review will be on the different memory processes that aggregate auditory experience into functional knowledge, and how these processes support category acquisition. Given that instability in speech sound representation has been linked to developmental language-based disorders such as dyslexia (Nation and Snowling, 1998) and Specific Language Impairment (Joanisse and Seidenberg, 2003), understanding of the memory encoding processes that impact phonetic acquisition may provide specific predictions for how disruptions manifest in behavioral symptoms, leading to the potential loci of breakdown or deficit.

In the present discussion, we review the theoretical models of category representation, arriving at the conclusion that two distinct cognitive processes support the perception of phonological categories: selective attention to certain features within the acoustic signal, and the recollection of a context within which those features are relevant. We argue that two types of learning are involved in the acquisition of categories: listeners must develop perceptual automaticity for selectively attending to familiar acoustic-phonetic features, and they must encode the context that dictates the relevance of these features. We then review selected literature on memory consolidation in auditory/perceptual skill and lexical form acquisition. Through this discussion, we illustrate that both types of learning are supported by memory encoding processes that take place during sleep.

## MEMORY IN MODELS OF SPEECH SOUND CATEGORY REPRESENTATION

Category acquisition is subject to the lack of invariance problem, in that “categories” represent our relatively constant perception of a signal that is subject to acoustic variability according to phonetic context, environmental conditions, and speaker idiosyncrasy (Nygaard et al., 1994; Nygaard and Pisoni, 1998; Allen and Miller, 2004; Eisner and McQueen, 2005; Kraljic and Samuel, 2005). The construction of a new category representation therefore requires the abstraction of information away from the individual acoustic events, such that knowledge of phonetic structure can be applied generally to novel tokens, new talkers, and different phonological contexts. Models of speech processing posit different theoretical constructs for how category knowledge is represented; however, they share in common the assumption that perception is influenced by experience (Kuhl, 1991; Iverson and Kuhl, 1995; Goldinger, 1998). This section will review how selected models of spoken language processing conceptualize phonetic categories, and how acoustic tokens might enter long-term store in order to exert an influence on online processing.

Attempts to resolve the lack of invariance problem must account for our propensity to attend specifically to the details of the acoustic signal that reflect features signaling a change in category. One framework, which posits the storage of idealized phonetic “prototypes,” is a popular construct by which to discuss the representation of speech sound categories (Iverson et al., 2003; Guenther et al., 2004; Athos et al., 2007). The notion of prototypes is realized by appropriating the generalized context model (GCM; Nosofsky, 1986), which allows the consideration of acoustic-phonetic features as dimensional structures within a perceptual space. Within this framework, language experience is thought to decrease the distance between acoustic tokens in the vicinity of prototypes, such that they behave as attractors that perceptually “pull” in acoustic items that fall within its boundaries [Native Language Magnet (NLM) model, Kuhl, 1991; Iverson and Kuhl, 1995]. Prototypes offer a potential solution to the lack of invariance problem by mapping many tokens onto one category, such that only one token, the prototype, is stored in memory for each phonetic category. While this is advantageous from the standpoint of efficiency in category retrieval, certain behavioral phenomena have prompted current iterations of magnet theory to consider the emergence of attractors as a consequence of statistical regularity in the input (Kuhl et al., 1992; Maye et al., 2002, 2008). The notion that one distribution in perceptual space reliably maps to one prototype makes it more difficult to account for context-dependent adjustments to the phonetic category boundary. For example, listeners adjust the perceptual boundary in the /s/ - /f/ continuum to the specific speaker after a period of exposure to the speaker’s particular pattern of articulation (Norris et al., 2003). Furthermore, listeners’ accommodations for speaker idiosyncrasy appear to be different for spectral and voice onset time (VOT) contrasts (Kraljic and Samuel, 2005). In addition, despite adults’ initial difficulty in perceiving non-native categories (Best, 1994; Iverson et al., 2003), perceptual training results in improved discriminability of non-native contrasts (Golestani and Zatorre, 2009; Swan and Myers, 2013). Under the prototype framework, this suggests that relatively little experience with non-native sounds can establish prototypes for novel categories, calling into question the amount and type of exposure that is necessary in order for categories to form.

An alternative is to consider a many-to-many mapping, such that each speech event is stored in memory as an individuated representation (Exemplar theory: Medin and Schaffer, 1978; Goldinger, 1998; see Pisoni and Levi, 2007 for review). Under this view, category identity is assigned through mapping the token to the category of similar sounds within previously encountered events. The traditional notion of a single prototypic exemplar acting as a perceptual magnet is replaced with the topography of perceptual space that is warped by the coherent convergence of past exposure to native exemplars. Thus, a prediction of how a non-native token will be perceived would not be determined by the relationship between the token and the prototypical exemplar, but by the probability by which the closest previously encountered exemplar belongs to a particular category. Similarly, statistical/distributional accounts (Pierrehumbert, 2003; Maye et al., 2008; Feldman et al., 2009) allow for episodes, over time, to create valleys in perceptual space without the provision of context. In this sense, these accounts are not wholly incompatible with NLM, in that a hypothetical token possessing the “average” of the featural values of all previously encountered exemplars in a given category would be in essence the category prototype. The difference between these perspectives is the nature of what is represented in memory: whether one prototypical exemplar of a category is stored as a perceptual attractor, or if an attractor emerges from the amalgamation of every experience individually stored. Furthermore, by exemplar-based and distributional accounts, the literal existence of “categories” is rendered unnecessary, as categorical perception may be epiphenomenal to the lexical and indexical cues that are stored in the representations of individual speech events.

The many-to-many mapping viewpoint thus offers a solution to the lack of invariance problem that accounts for stable percepts of “category” that also flexibly adapt to context. Experience, however, increases the number of previously stored encounters, increasing the number of tokens against which new encounters must be considered; in other words, the literal storage of individuated exemplars may tax perceptual efficiency rather than facilitate it. Versions of the exemplar model address this issue through considering the reorganization of perceptual space as the modification of weights on the connections between tiers of information. For example, in Kruschke’s (1992) Attention Learning Covering Map (ALCOVE) model, the three-layer connectionist model architecture specifies stimulus dimension nodes that receive the input, a hidden layer in which every exemplar is represented by a single node, and category nodes at the output. The weights between the exemplar and category nodes are adjusted per trial by a learned association algorithm, while the connections between stimulus features and exemplars are adjusted by a selective attention algorithm such that “attending” to a particular feature during training will expand the perceptual distance along that continuum. Thus, the model’s mechanism of perceptual learning is to alter its degree of sensitivity to distinctions in features. This key innovation of the model in incorporating selective attention allows for active cognitive processing during the perceptual event to mediate the gradation of impact that experience has on the underlying perceptual topography. In this sense, while exemplars are the currency upon which the model is built, the model itself results in a warping of perceptual space, such that processing new tokens is achieved by active competition and cooperation between the increased automaticity of bottom-up processing and a set of static category boundaries.

There remains, however, an outstanding issue that is common to both many-to-one mapping and many-to-many mapping approaches that needs to be addressed. This is the problematic assumption that the perception of any exemplar is pre-determined, prior to the perceptual task and regardless of context, by the relationship between the stimulus and the listener’s topography of underlying perceptual space (Nusbaum and Magnuson, 1997). This assumption breaks down when we consider the contribution of listener expectation. For example, the listeners’ expectations about the speaker’s gender alter their perception of an ambiguous fricative along the /s/ - /*∫*/ continuum (Strand, 1999). Furthermore, prior knowledge of the message content can render an otherwise unintelligible message delivered by sine-wave speech intelligible (Remez et al., 1981). Finally, in a speeded target-monitoring task, Magnuson and Nusbaum (2007) found that participants instructed that they will hear two different talkers responded more slowly on a speech identification task containing alternating voices relative to participants who were given no such instruction, despite both groups receiving acoustically identical stimuli. Thus, the listener’s expectations about the signal prior to exposure appears to mediate signal processing, which is incongruous with the notion that perception is determined only by the features of the incoming signal interacting with the pre-task topography of perceptual space. Memory of previous experience (one vs. multiple talkers in this case) appears to set up listener expectations that constrain the set of features to which he/she selectively attends.

In concert with this idea, the Attention-to-dimension approach (A2D; Francis et al., 2000; Francis and Nusbaum, 2002) assumes no default (pre-task) warping of perceptual space, and points to active selective attention giving rise to the differential weighting of acoustic features as relevant for the completion of a perceptual task. To illustrate, Francis et al. (2000) found that listeners given the same set of acoustic cues, but different category labels by which to assign them, learned to attend to different features in the signal. The authors suggest that the top-down input of category, which in this case was provided, inform the listener’s allocation of selective attention to different features for completion of the task. To reiterate, in contrast to exemplar accounts, A2D posits that category identity is not determined by the information contained within the acoustic signal, but rather the listener’s active cognitive processes acting upon the input. This distinction allows for listeners to receive identical acoustic inputs and yet differ behaviorally in a perceptual task according to their expectations about the signal (e.g., Magnuson and Nusbaum, 2007). This is a qualitatively different role of selective attention with respect to ALCOVE, in that attention’s primary role is not to make lasting changes to perceptual space, but to give task-specific guidance for perception. This account better explains how adults with no prior foreign language experience can learn to perceive non-native contrasts quickly with training. Specifically, this framework suggests that while increased practice may reduce attentional effort, there is no default pre-task bias that determines perception. Rather, we rely on memory to provide a representation of context (such as the lexical context, speaker, or category label) during online processing that constrains the acoustic dimensions along which we allocate attentional resources.

This idea is further consistent with Goldstone’s (1994) solution for category formation in visual perception: while the physiological constraints of signal perception may somewhat guide attention along certain dimensions, “category” is a malleable, abstract construct that provides top-down context for guiding attention along certain features. In support of this idea, Goldstone (1995) demonstrated that participants’ judgments on the saturation of a hue were mediated by the object on which the color was perceived (i.e., the redness on a typically red object is judged more red than the identical redness on a typically yellow object), indicating that one’s abstract representation of an object’s typical color category affects perception of the featural (color intensity) value. In speech terms, this allows language novices with little experience to have functional boundaries for novel categories, and each encounter with a new token will refine their knowledge about the context-dependent location of these boundaries.

In order to connect perceptual learning to the memory literature, we discuss what is learnt in terms of the memory system active during learning. We emphasize, however, that the conceptual underpinnings resonate with existing theoretical models of category representation. Kruschke’s model posits that two types of information are considered relevant to perception; similarly, we propose that the acquisition of novel categories involves both the encoding of a context (talker/lexical/category label, etc.) that specifies the acoustic-phonetic features of interest (exemplar theory), and the reduction of effort/increased automaticity in attending to values along those continua (A2D). Furthermore, behavioral assessments of these two types of learning are likely to engage different memory systems. Specifically, automaticity of attention to features is proposed to be enhanced by the procedural memory system, while the top-down source of influence (context-specific location of category boundaries), are predicted to be encoded by the structures that support declarative memory formation. Before highlighting the rationale for this distinction, the discussion will first turn to a focused review of memory consolidation as it relates to speech-related learning, and the roles of sleep on procedural and declarative memory. In combining these insights, we will arrive at a means by which we can test potential loci of deficits in individuals who struggle to form stable representations.

## OVERVIEW OF MEMORY CONSOLIDATION

As indicated above, memory processes are implicit in all models of category acquisition. In order to understand the nature of the representation that is formed, it is important to understand how phonetic details enter long-term memory through memory consolidation processes. The term “consolidation” was coined by Müller and Pilzecker (1900), who observed that the recall of a list of non-sense syllables were more accurate in participants tested following an interval of time, in comparison to those who were tested shortly after learning. The past half-century of research in the time course of selective changes to the memory trace has highlighted at least two different types of consolidation processes that take place (Dudai, 2004). These events are the local changes to neural structure immediately (minutes to hours) following the learning event (synaptic, early, or short-term consolidation), and the slow (days to months) reorganization of the neural network reflecting long-term storage (systems, late, or long-term consolidation). Therefore, “consolidation” is a collective reference to the stages of information processing that result in either local or systemic neural changes, with qualitative effects on the recollected memory trace observable in behavior. In relation to the acquisition of novel phonetic categories, we propose that synaptic consolidation precipitates perceptual automaticity, while systems consolidation results in the context representation that specifies the acoustic-phonetic features of interest.

### SYNAPTIC CONSOLIDATION

Synaptic consolidation is proposed to be the mechanism underlying enhanced procedural learning (Vertes, 2004), and therefore tied to acquiring perceptual automaticity. Following an inducing (perceptual) event, early long-term potentiation (LTP; Bliss and Lømo, 1973), in conjunction with late-phase LTP, or synaptic consolidation, stabilizes the initial expression of the memory trace. LTP is a persistent enhancement of synaptic potential immediately following an inducing event that allows weaker inputs to also strengthen synapses when activated. During late-phase LTP, or synaptic consolidation, protein synthesis yields structural changes to active dendritic spines (Bramham and Messaoudi, 2005), crucial to memory maintenance beyond the first few minutes to hours. A standard model of synaptic consolidation (Bliss and Collingridge, 1993; Dudai, 2004) summarizes that processes associated with early LTP, lasting immediately to a few hours following induction, yield local changes that decay in time. Long-term changes to synaptic strength further require induction by a strong stimulus that triggers protein synthesis, resulting in stabilization of the active synapse by enlarging the dendritic spine and chemically binding the active pre-/post-synaptic terminals. The resultant synapse is more efficient in signal transmission and resistant to modification. Below, we discuss the evidence that sleep promotes latent synaptic consolidation; behavioral correlates of sleep-mediated synaptic consolidation includes the enhanced ability for explicit recall of new information (e.g., Rasch et al., 2007), or enhanced proficiency in an acquired skill (e.g., Walker et al., 2003) including auditory perceptual skill (Brawn et al., 2010). We argue that similar processes promote perceptual automaticity in speech.

### SYSTEMS CONSOLIDATION

We propose that distributed (and therefore generalizable) context representations (such as the lexical context, speaker, environment, etc.) that provide top-down guidance for online perception results from the abstraction of episodic information during systems consolidation (Davis et al., 2009). Systems consolidation is a process by which new memories are integrated with pre-existing information, and is primarily associated with hippocampal-encoded memory. The account of systems consolidation is based on a theoretical argument for the necessity of separate structures for temporary vs. permanent storage of memory (McClelland et al., 1995). During an experience, the hippocampus is suggested to automatically capture cortical activity and store an index to that pattern as a minimal representation that can be later reactivated for the recollection of an event (e.g., McNaughton and Morris, 1987; O’Reilly and Rudy, 2001). However, the hippocampal architecture is unlikely to provide a solution for permanent memory storage (Marr, 1971). McClelland et al. (1995) illustrated the necessity of separate structures for the temporary vs. permanent storage of memory, by revisiting Rumelhart’s (1990) simulations of semantic learning. The network implements learning as the unsupervised discovery of shared structures following exposure to various exemplars (as denoted by a set of features) of target concepts. Rapid, focused learning by such networks lead to catastrophic interference, such that new information overrides conflicting pre-existing information. This is resolved by modifying the training procedure to gradual, interleaved learning between trials of different concepts with shared features. During the capture of an experience, rapid encoding of sensory information does not allow for slow integration with pre-existing knowledge: thus, the authors proposed that the hippocampus gives temporary storage to rapid encoding of episodes, which are slowly integrated with the cortex off-line for long-term storage. Semantic knowledge may then arise from the shared structures that emerge in the cortex over a lifetime of experience. In speech learning, systems consolidation may give rise to context-independent, abstract category knowledge that allows listeners to recognize sounds in unfamiliar lexical items or spoken by novel talkers.

By comparison to synaptic consolidation, systems consolidation is less well understood. This is in part because systems consolidation is a slow process, whereas synaptic consolidation for a new trace is generally accepted to complete within 24 h. Furthermore, systems consolidation is difficult to establish in behavior in that performance must somehow assess the state of the memory trace as either in an integrated or non-integrated state. Candidate behavioral (e.g., Dumay and Gaskell, 2007) and physiological observations (e.g., Ji and Wilson, 2007) that potentially reflect this off-line transfer of information between short and long-term storage is discussed in the following section.

### THE RELEVANCE OF SLEEP TO SPEECH-RELATED LEARNING

There is evidence to suggest that sleep supports various aspects of language acquisition (see Gomez et al., 2011 for review). For example, naps have been found to support the abstraction of syntactic dependencies of an artificial language (Gómez et al., 2006), and in the retention of these abstractions 24-h post-exposure (Hupbach et al., 2009). Exposure to speech sound stimuli during sleep has been further demonstrated to lead to significant change in preattentive discrimination in newborns (Cheour et al., 2002). In the wider consolidation literature, there is debate as to the necessity of sleep to memory consolidation, in part due to the confusion in terms; for example, consolidation has been linked to stabilization against interference (Brashers-Krug et al., 1996), skill enhancement (Karni et al., 1994), integration (Tamminen et al., 2010), or selective forgetting (Saletin et al., 2011). Interpretation of consolidation effects as dependent on a period of time spent in sleep state, or as merely the result of passage of time in either wake or sleep, largely depends on the behavioral metric used. Below, we address how these differences in consolidation effects reflect learning that engages different memory systems according to the type of information acquired or the manner in which it is acquired (Tulving, 1985; Nissen and Bullemer, 1987; Squire, 1992). This is important to keep in mind, as the task used to measure consolidation in phonetic learning will affect the presence and nature of effects that are specific to mediation by sleep. To specify, “procedural” learning is considered as the implicit acquisition of skill (Doyon, 1997), and “declarative” learning indicates acquisition of information that can be explicitly recalled (O’Keefe and Nadel, 1978; O’Reilly and Rudy, 2001). It has been suggested that rapid-eye movement (REM) sleep preferentially consolidates procedural memory, while non-REM/slow-wave sleep (SWS) promotes consolidation of declarative memory (Marshall and Born, 2007). This review will discuss the evidence for these claims, in relation to the kinds of learning that are relevant to our discussion of phonetic learning.

#### CONSOLIDATION DURING REM SLEEP AND AUDITORY SKILL LEARNING

Learning-induced perceptual automaticity is considered here as analogous to the acquisition of an auditory/perceptual skill. There is a wide literature documenting the sleep-mediated improvement of procedural memory (see Smith, 2001; Stickgold, 2005 for review). A period of sleep, as compared to a comparable period of wakefulness, has been observed to lead to improved visual perceptual discrimination (Stickgold et al., 2000a,b), motor skill learning (Walker et al., 2002, 2003; Fischer et al., 2005), and auditory discrimination (Brawn et al., 2010). The memory literature often discusses auditory/perceptual skill acquisition as an example of procedural learning, in that this type of learning is measured by the implicit improvement in skill/task performance rather than in the declarative recall of experience (e.g., Gaab et al., 2004; Walker and Stickgold, 2004; Margoliash and Fenn, 2008). Within this literature, convergent evidence points to a time-dependent stabilization of a learned auditory skill, followed by a sleep-mediated increase in perceptual automaticity. For example, Gaab et al. (2004) investigated the retention of pitch learning in the 24 h following initial training at three time points post-training (immediate, 12-h post, and 24-h post). The authors found that the participants, regardless of when they were trained, showed latent improvement in discrimination only after the post-sleep interval. Similarly, Brawn et al. (2010) found that adult starlings trained on discrimination of segments of natural birdsong improved in performance following an interval containing sleep, whereas no improvement was observed following comparable intervals of wake state. On the other hand, Gottselig et al. (2004) measured improvement after training in participants grouped in one of four post-training conditions: no break (NB; immediate post-test administered after training), restful waking (RW; training-to-test interval spent awake in a dark, quiet room), busy waking (BW; training-to-test interval spent watching a film), and sleep (training-to-testing interval spent napping). While the authors interpret their findings as a lack of sleep-dependent effects, an alternative interpretation for their reported *p*-values is that the differences between the sleep group and the NB/RW groups respectively appear to be approaching significance, but not between NB and RW. In other words, performance is stable across time in the absence of interfering information, but task *improvement* without additional training is observed only after sleep.

Enhancement of procedural/perceptual skill has been tied to REM sleep. The REM stage in sleep has been found to be longer in duration and more frequent in sleep immediately following procedural training (e.g., Smith, 1985; Smith and Lapp, 1991). REM sleep deprivation has been linked to selective impairments in implicit learning, while leaving performance on declarative tasks intact (Smith and Kelly, 1988; Conway and Smith, 1994; Smith, 1995). Karni et al. (1994) demonstrated that selective disruptions to REM sleep, but not to non-REM sleep, prevent improvement on a learned visual perceptual task. Taken together, these studies have been taken as evidence that REM plays a consolidation role specific to procedural learning (see Stickgold et al., 2000a,b for review). Furthermore, Schwartz et al. (2002) established using fMRI that the neural correlates of sleep-mediated improvement in monocular visual texture discrimination are localized changes in functional connectivity in the primary sensory cortex. The authors concluded that the mechanism of implicit perceptual learning involves strengthening of local connections in the sensory cortex engaged during the wake-state activity. In other words, the mechanism underlying the perceptual skill improvement appears to be supported by processes similar to latent synaptic consolidation (Vertes, 2004).

Revisiting the concept that perceptual training for speech involves learning to selectively attend to relevant acoustic features (Francis et al., 2000), the initial acquisition phase of the novel phonetic information would imply an effortful attentional shift to the features that are distinctive for the novel category. Synaptic consolidation, in increasing the efficiency of neural transmission through structural change, would thereby reduce the amount of stimulus signal necessary to elicit a response (Bramham and Messaoudi, 2005), and the corresponding effort required to selectively attend to relevant features (Atienza et al., 2004). The previous discussion on the consolidation of procedural/implicit learning during REM sleep suggests that improvement in perceptual skills reflect latent synaptic consolidation. This interpretation is consistent with Atienza et al. (2004), who reported on the post-training changes to the event-related potential (ERP) evoked during auditory discrimination. They found that while sleep deprivation did not prevent the enhancement in magnitude of mismatch negativity (MMN) response over time, an automatic shift of attention to stimuli, as measured by the P3a component, failed to develop in the sleep-deprived condition. The authors suggest that the role of sleep for auditory skill learning is to reduce the amount of effort required for attentional shift to learned stimuli, thereby increasing perceptual automaticity. Thus, in speech, the perceptual discrimination of novel speech tokens is likely to be similarly strengthened during REM sleep following perceptual training.

#### CONSOLIDATION DURING NON-REM SLEEP AND NOVEL WORD FORM LEARNING

In contrast to the REM sleep-mediated enhancement of perceptual automaticity, we propose that the representation of a context (such as lexical context, information about the speaker, category label, etc.) that guides the acoustic-phonetic features of interest is mediated by the structures associated with the declarative memory system. Qualitative changes to declarative memory are associated with neural activity observed during non-REM sleep. Evidence that sleep plays a role in systems consolidation of hippocampal memories comes from the observation of neural “replay” activity (Wilson and McNaughton, 1994; Skaggs and McNaughton, 1996; Ji and Wilson, 2007) in hippocampal place cells (O’Keefe and Dostrovsky, 1971) of the sleeping rat. By taking unit-cell recordings in rats trained to sleep immediately before and after spatial exploration, Wilson and McNaughton (1994) observed that hippocampal cells that fired together during wake-state experience had a significantly higher likelihood of firing together during post-task, but not pre-task, SWS. In establishing “frames” of place cell firing sequences, Wilson and McNaughton (1994) further observed that hippocampal activity during sleep was “replays” of exact neural sequences active during the pre-sleep spatial exploration. Building on this, Ji and Wilson (2007) established that hippocampal replay events are temporally coordinated with cortical replays in the visual cortex during SWS, indicating that the hippocampal–cortical dialog during sleep reflects co-activity during wake-state experience. In addition, the same replay events were absent in the same animals sleeping ~24 h post-run, indicating that memory reactivation during sleep reflects wake-state activity immediately prior to sleep. These results are consistent with models of systems consolidation that posit an off-line transfer of episodic information from the hippocampus to long-term storage (McClelland et al., 1995), suggesting that the hippocampal–cortical temporal coordination reflects this transfer.

Following this rationale, several lines of research have worked to establish a link between non-REM sleep and systems integration of declarative memory. Considerable behavioral support for the role of sleep in the transfer and integration of information is provided by the novel word learning literature (Gaskell and Dumay, 2003; Dumay et al., 2004; Dumay and Gaskell, 2007; Tamminen and Gaskell, 2008; Davis et al., 2009; Tamminen et al., 2010). These studies share the premise that word recognition undergoes a process of activation of all possible candidate items in the mental lexicon, followed by the determination of the most likely lexical candidate for the input based on the information that unfolds over time (e.g., Cohort model, Marslen-Wilson, 1987; TRACE, McClelland and Elman, 1986). Words that are simultaneously activated are said to be in competition; task performance is therefore mediated by word frequency and phonological neighborhood density (Neighborhood activation model, Goldinger et al., 1989; Luce and Pisoni, 1998): high frequency words, and/or words occurring in a sparse phonological neighborhood, are recognized more quickly than low-frequency words in a dense neighborhood. For phonological sequences to be considered as having “word” status, they must exert similar effects of competition on existing words in the lexicon. Gaskell and Dumay (2003) trained participants on the phonological structure of novel items such as *cathedruke*, thereby introducing a competitor to a pre-existing lexical item such as *cathedral* in a sparse phonological neighborhood. Following training, the new item facilitated faster response times in lexical decision to their competitor English words (e.g., “cathedral”). However, 1 week following the training, response times in lexical decision on the pre-existing words were significantly slowed in comparison to performance on the day of training. The authors claimed that sometime during that interval, the novel words were inserted into the mental lexicon such that they became active competitors to their phonological neighbors. In a similar paradigm assessed over 24-h, Dumay and Gaskell (2007) further demonstrated that lexical competition effects emerge following the first overnight interval, but not during a comparable daytime interval. Furthermore, these lexical competition effects that emerge over the initial 24 h are still present 8 months following the initial acquisition, indicating that the insertion of a phonological string into the mental lexicon reflects a lasting change in representational status (Tamminen and Gaskell, 2008).

Since then, overnight emergence of lexical competition has been confirmed through variations on this paradigm, such as through employing semantic training on the novel words (Dumay et al., 2004), investigating effects of lexicalization on speech segmentation (Dumay and Gaskell, 2012), or effects measured through pause detection paradigms (Dumay et al., 2004). The degree of increase in reaction time (interpreted as degree of lexical integration) has been found to correspond to duration of SWS (Tamminen et al., 2010), and furthermore the relationship of the learned novel semantic features to semantic feature neighborhood density has been shown to affect subsequent non-REM sleep architecture (Tamminen et al., 2013). Davis et al. (2009) further obtained neural correlates of the “lexicalization” effect through fMRI imaging. The authors trained novel and existing words on 2 days, and recorded participants’ neural responses to novel familiar unconsolidated, novel unfamiliar, novel consolidated, and existing words. The authors found that the initial acquisition of novel words appeared to recruit the hippocampus, and while the pattern of cortical activity for newly learned words was most similar to unfamiliar words on day 1, it came to resemble activation for existing words on day 2. The interpretation of the collective works has been that novel word forms integrate with the mental lexicon overnight through off-line interleaving of novel information (Davis and Gaskell, 2009), consistent with the *Complementary Systems Account* of learning (McClelland et al., 1995). It has been suggested that the repeated reactivation of hippocampal memories during neocortical slow oscillations result in the redistribution of episodic information to cortical networks, leading to a reorganization of abstract knowledge (Rasch and Born, 2013). Taken together with the evidence on word learning, we propose that the analogous role of systems consolidation in speech learning is to abstract and integrate novel phonetic tokens into the pre-existing phonology, such that abstract knowledge may exert top-down influence on perception.

#### DISSOCIATION IN SLEEP STAGES REFLECTS SYNAPTIC VS. SYSTEMS CONSOLIDATION

The above discussion on sleep highlights the dissociation between non-REM sleep as preferentially responsible for declarative memory consolidation, and REM sleep for procedural memory consolidation. Before moving to our discussion on phonetic learning, it is important to first clarify that the declarative/procedural dissociation in consolidation effects is a historical convention of the wider memory literature (see Smith, 2001 for review) that current theories now consider an artifact of the task used to measure sleep-mediated effects (Diekelmann and Born, 2010). To illustrate the distinction, Cairney et al. (2011) reported that the abstraction of statistical regularities in tone sequences emerged after sleep as a function of SWS quality, even though the task tapped an implicitly learned still. Durrant et al. (2013) examined the neural correlates of implicitly learned statistical regularities using fMRI, and found that the duration of SWS corresponded to stronger striatal, and weaker parahippocampal, responses following sleep. Taken together with the behavioral evidence, the authors claim that abstractions for statistical regularities involve information transfer from the hippocampus to the striatum during sleep. Thus, it appears that the effects of systems consolidation (abstraction) are not limited to learning by the declarative system.

Therefore, the alternative is to consider the differential effects observed during different sleep stages as attributable to synaptic vs. systems consolidation. Diekelmann and Born (2010) propose a two-step model of active, sequential consolidation during the REM–SWS cycles of sleep to illustrate this concept. Specifically, the authors suggest that spindle activity during phase 2 non-REM sleep initiates a reorganization of the cortex, followed by the reactivation of selective wake-state experiences through coordinated hippocampal–neocortical replay in SWS that result in distributed representations. During the subsequent phase of REM, regions of the brain are proposed to undergo localized synaptic consolidation, enhancing the automaticity of signal processing within these regions. Thus, the association of REM/non-REM sleep with improvement in procedural vs. declarative tasks is thought to be reflective of the consolidation type (synaptic vs. systems).

Our predictions for how selective disruptions to specific sleep stages affects perceptual learning is built upon our assumptions for how Diekelmann and Born’s (2010) two-step model applies to the two types of learning that we have highlighted as relevant in acquiring categories. It is therefore important to keep in mind that consolidation effects to be expected for phonetic learning depend on the design of the training and the task used to measure learning: specifically, which type of consolidation (synaptic, systems, or both) is likely to improve performance on a particular task. For the purposes of discussion, however, we will continue to refer to the procedural/declarative distinction to align the memory system with tasks that primarily engage each system. Specifically, perceptual learning measured by tasks that rely on acquired auditory skill, we will refer to as “procedural,” while tasks that measure the explicit recall of information, we will refer to as “declarative.” This is not to imply that we believe these memory systems to act independently in encoding phonetic information, but rather that the tasks used to assess perceptual learning differentially manipulate the relative reliance on auditory skill or explicit recall.

## PREDICTIONS FOR THE ROLE OF SLEEP IN PERCEPTUAL LEARNING OF SPEECH

Few studies have directly investigated the role of consolidation in perceptual learning relating to speech, with some studies suggesting a role of sleep, and others not. For example, lexically guided perceptual adjustments to the categorical boundary along a /f/-/s/ continuum have been found to emerge immediately, and to remain stable over the 24-h experiment period (Eisner and McQueen, 2006), suggesting no performance change associated with sleep or the passage of time. In contrast, in a syllable-identification-in-noise task, Roth et al. (2005) showed either no gains or degraded performance up to 5 h post-training, but significant improvement 6–12 h after training regardless of whether or not the interval between training and retest contained sleep. Thus, some training-induced changes in speech perception appear not to derive benefit from sleep. In contrast, Fenn et al. (2003) observed a different pattern, in which changes to performance over time depended on when the training took place in relation to sleep. Their task was to identify words presented in synthetic speech; no word was used more than once, such that training required that listeners generalize the mapping of synthetic speech to pre-existing phonology. For those trained at 9 p.m., the overnight between-session interval appeared to have a stabilizing effect against degradation in performance. In those trained at 9 a.m., performance appeared to degrade during the day and then was restored overnight. These studies therefore suggest different roles for consolidation over time and sleep depending on the perceptual measure used.

This inconsistency may be potentially resolved by considering the phonetic information contributing to the pre-existing phonology by the training task. In both the studies by Eisner and McQueen (2006) and Roth et al. (2005), the perceptual adjustments are made within pre-established category boundaries. In other words, neither study requires any reorganization of the phonological system, or any new information to be added to inform pre-existing category structure. In contrast, the mapping task in Fenn et al. (2003) required a systemic re-definition of representations to include synthetic speech as acceptable exemplars. Thus, the sleep-mediated changes to behavioral performance may be more salient in tasks that benefit from the addition of phonetic information to, or systemic reorganization of, the established phonology. This interpretation is supported by a recent study by Fenn et al. (2013), comparing the sleep-mediated effects of rote vs. generalized training of mapping synthetic speech to native phonology. They found that the rote-trained group, trained on a closed set of the same 20 words, improved after sleep in the trained words only, but the generalization-trained group, trained on unique stimuli for every trial, demonstrated significant improvement on novel words after sleep.

**FIGURE 1:**
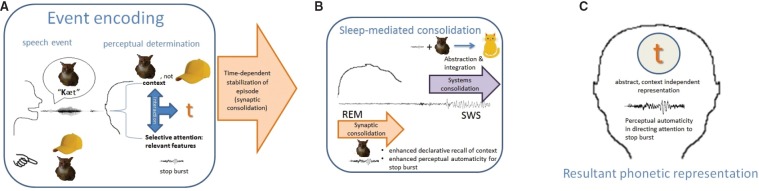
**(A)** Perception is determined by the competitive and cooperative interaction between selective attention to the relevant feature, given the event context. In this hypothetical situation, the reference context specifies the feature of interest to be the stop burst, to disambiguate /Kæt/ from /kæp/. **(B)** Systems consolidation abstracts the information away from the event context and integrates it with pre-existing knowledge. Synaptic consolidation strengthens established connections, thereby facilitating declarative recall of the event context and enhancing the automaticity by which attention is selectively tuned to the feature that was relevant during the event (i.e., stop burst). **(C)** The resultant phonetic representation of /t/ is contextually flexible. Shift of attention to selectively attend to the stop burst is enhanced, independent of context.

Applying the above insights, we can expect several sleep-related changes to performance on tasks that measure the encoding of novel phonetic information (see Figure 1). In the procedural memory system, synaptic consolidation may promote automaticity in the implicitly acquired shift of attention to selective features, leading to enhanced performance on perceptual discrimination of novel speech sounds. In addition, synaptic consolidation in the declarative memory system may lead to improvement in the explicit recall of the category label, resulting in faster identification. Systems consolidation on the other hand, would promote the abstraction and integration/reorganization of novel phonetic information within the pre-existing mental phonology, such that the identification of novel phonetic tokens are no longer bound to episodic information such as the specific talker encountered in the learning event. We would expect to observe this in the generalization of novel phonetic information to the identification of the newly learned phonetic items spoken by a different talker, or occurring in a different phonetic environment, with respect to tokens used during learning.

To reiterate, this account predicts that the two behavioral tasks often used to measure phonetic learning, discrimination and identification, rely in different degrees on information encoded by two different memory systems. Improvement in perceptual discrimination may rely on learned selective attention to relevant features, which, as an implicitly acquired perceptual skill, is primarily mediated by the procedural memory system. The task of identification, as an explicit recall of a category label, is mediated by the declarative memory system. This claim is supported by differences recently observed in our lab on the sleep-mediated effects on task performance between discrimination and identification tasks (Earle and Myers, 2014). Specifically, we found that passive exposure to conflicting phonetic information following the perceptual training of non-native consonants prevented the overnight improvement of perceptual discrimination, consistent with retroactive interference effects observed in the sleep-mediated consolidation of procedural learning in other domains (e.g., Walker et al., 2003). The interference condition, however, did not prevent improved identification of the trained non-native phonetic tokens over time, suggesting that the information recalled for identification is not susceptible to the same interference effects that are detrimental to perceptual discrimination. This robustness of performance on the identification task relative to the discrimination task may be due to the contribution of episodic information through the engagement of the declarative memory system during the identification task. This interpretation further predicts that the generalization of the trained speech sounds across different talkers and different acoustic contexts will be more likely to be observed in a task involving explicit recall, such as identification, over an application of implicit skill, such as discrimination.

The task dissociation furthermore reflects the two sources of information that are highlighted above as necessary for efficient online spoken language processing: the implicitly acquired perceptual automaticity in directing attention selective to relevant features in the signal, and the context in which features are evaluated as similar/dissimilar. Learned selective attention, as might be preferentially improved through synaptic consolidation, underlies changes to behavioral performance in discrimination. The encoding of the context that specifies category boundaries, we propose is bound to the declarative memory system; the category boundaries are initially tied to the episodic capture of the experience, followed by an off-line abstraction away from the episode-specific details.

## PREDICTED CONSEQUENCES TO HABITUAL DISRUPTION IN SLEEP

The current discussion further yields specific predictions for how habitual sleep disruptions may affect the encoding of phonetic information. This topic is motivated by reports in the developmentally language-impaired population of paroxysmal activity during sleep (Duvelleroy-Hommet et al., 1995; Picard et al., 1998), along with abnormal development in brain structures associated with the procedural memory system (Procedural Deficit Hypothesis; Ullman and Pierpont, 2005). Language impairment in autism has furthermore been linked to epileptiform activity observed during sleep (Patzold et al., 1998; Ballaban-Gil and Tuchman, 2000); furthermore, the onset of language regression in Landau–Kleffner is associated with epileptic activity observed during SWS (Massa et al., 2000; McVicar and Shinnar, 2004). As epileptic activity implies potential structural damage, it is not the intent of the current review to argue that the language impairments in these populations are directly caused by disruptions to sleep. Rather, the following discussion will outline the potential consequences to phonological stability that we might expect from habitual disruptions to the discrete structures or processes involved in building phonological representations.

**FIGURE 2:**
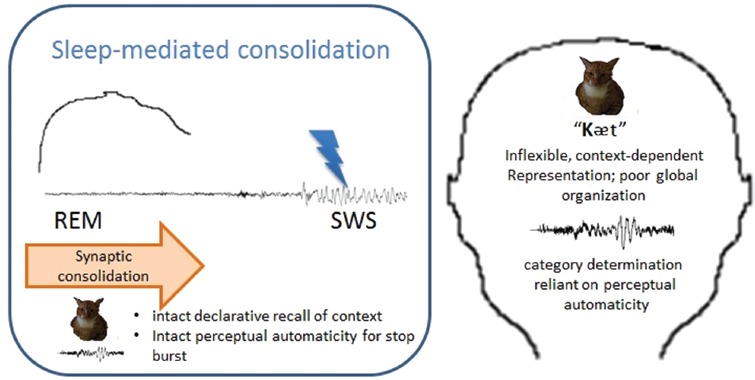
**The habitual disruption of SWS would prevent the abstraction and integration of information, such that spoken language processing would rely on the relatively intact perceptual automaticity and local connections made during the initial encoding event; in other words, while perceptual automaticity remains intact, the representation of contextual information will be dependent on the details of the encoding episode.**

First, structural abnormality in the procedural memory system leads to a compromised ability to build speed/automaticity in selectively attending to features that are relevant in the acoustic signal. This may lead to slowed and/or effortful signal processing, increasing the cognitive demands placed on online comprehension of spoken language (see Figure 2). In contrast, declarative systems mediate explicit recall of episodic and semantic memory; thus, structural/connectivity differences in the declarative system structures, may result in deficits in the encoding/recall of episodic details of the signal such as talker features and in the abstraction of these details that underlie category structure.

**FIGURE 3:**
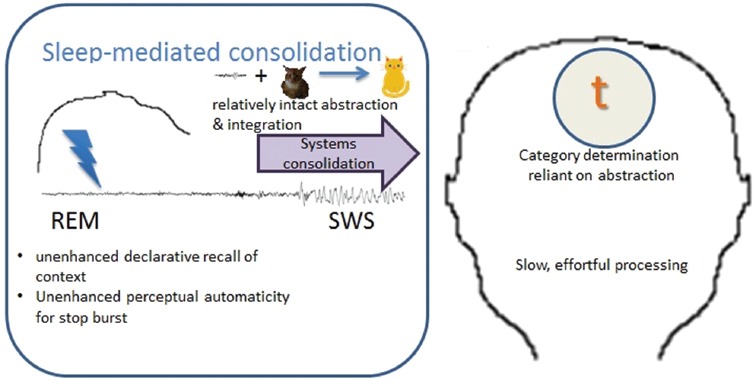
**The habitual disruption of REM sleep would prevent the enhancement of declarative recall of both the contextual information and the automaticity in directing attention to the feature that was relevant during the perceptual event (i.e., the stop burst, in this hypothetical situation).**

In considering a functional abnormality, we might expect that selective habitual disruption to REM sleep may prevent both the enhancement of perceptual automaticity and of episodic recall. A selective habitual disruption to SWS, on the other hand, may prevent the formation of abstract categories and the transfer of information between systems, leading to poor global organization of categories with relatively intact local connections. Behaviorally, this may manifest in sensitivity to non-distinctive cues in the signal, without contextual or top-down knowledge guiding efficient organization of the input (see Figure 3). Examples of speech-specific difficulties for such a system may be in the comprehension of a degraded signal, such as over the phone, or in adverse conditions, such as in background noise. Furthermore, such systems may be less adaptable to accented or novel talkers. Finally, a structural or functional abnormality in either system can lead to impoverished representational quality overall, in compromising the cohesion between the two sources of input.

In conclusion, there is much work to be done on the role of sleep in phonetic learning. There are several outstanding issues highlighted here regarding the contribution of discrete memory encoding processes on speech perception. In defining the time course of typical encoding of novel phonetic tokens, we may begin to disentangle if/how these encoding processes are compromised in those with language symptoms concurrent to developmental disability.

### Conflict of Interest Statement

The authors declare that the research was conducted in the absence of any commercial or financial relationships that could be construed as a potential conflict of interest.

## Footnotes

1 We use “phonetic category” to describe the distribution of acoustic tokens which together are perceived as mapping to a phoneme in the listener’s inventory.
